# The movement of class: on occupation and everyday mobility patterns in the United States

**DOI:** 10.3389/fsoc.2025.1492785

**Published:** 2025-04-10

**Authors:** Karl Vachuska

**Affiliations:** Department of Sociology, University of Wisconsin-Madison, Madison, WI, United States

**Keywords:** class analysis, everyday mobility patterns, neighborhoods, occupation, social stratification

## Abstract

For much of the last century, class analysis has been a major area of sociology and has provided a critical lens through which scholars analyze social stratification. The attributes of certain class positions are of particular sociological interest given their impact on stratification and the possibility of greater inter- and intra-generational mobility. In this work, I explore one perspective of class analysis that has been neglected in the literature: everyday mobility patterns. As a result of the rising availability of rich cell phone data, everyday mobility patterns have become a popular data source for social science research. However, despite the clear theoretical relationship between everyday mobility patterns and class, little sociological research has connected these two concepts. The analysis, set in the United States, indicates that class—specifically, occupational class—is an extremely strong predictor of mobility patterns and that not all occupations are associated with the mobility patterns one might expect. The findings also indicate that certain occupations are disproportionally exposed to impoverished neighborhoods, and I thus theorize about the occupational attributes that matter most for everyday mobility patterns. I conclude by arguing that novel data sources have the potential to renew interest in class analysis.

## Introduction

Understanding the relationship between occupational inequality and everyday mobility patterns provides valuable insights into the dynamics of social stratification and urban inequality. While existing research has extensively examined occupational class through the lenses of education, income, and prestige, relatively little attention has been given to how occupation is associated with patterns of movement through the urban environment. Sociological research on everyday mobility patterns has grown rapidly in recent years with the advent of large-scale cell phone mobility, with research finding mobility patterns are important for predicting neighborhood outcomes (Levy et al., 2022). By examining these mobility patterns, this paper seeks to uncover how occupational class influences not only where people go but also the conditions they encounter, such as exposure to neighborhood poverty. This focus allows us to bridge the gap between occupational class analysis and urban mobility studies, offering a novel perspective on how social inequality manifests in spatial terms.

In sociology, a large body of class analysis scholars has explored what differentiates occupational classes from one another and what occupational attributes have the greatest implications for intra- and inter-generational mobility. Early research focused on the levels of education and income associated with specific occupations, assigning socioeconomic and prestige scores to different occupations based on these two traits (Duncan, 1961; Goldthorpe and Hope, 1972; Hodge et al., 1964; Featherman and Hauser, 1976). A long line of work identified that such occupational traits tended to correlate with one’s life chances and those of one’s children. However, more recent research has discouraged such simplifications of class, arguing that measures of class attainment must be specific to what is being analyzed (Hauser and Warren, 1997).

How best to group occupations, which are themselves groupings of distinct vocational roles, has been a topic of much debate. For Marx, class analysis divided occupations into broad macroclasses based on their relation to the means of production (Dahrendorf, 1959). Because these broad categories reflected an individual’s position within a capitalist division of labor, it was argued that this was the most salient stratum through which inequality manifested. However, subsequent research began to break down occupational classes into smaller categories, as it was observed that the same occupations tended toward the same perceived prestige across many countries.

Recent dialogue in the literature has concluded that a decline in occupational macro-class significance has occurred (Weeden and Grusky, 2012). Weeden and Grusky (2012) present the hypotheticals of “big-class,” “micro-class,” and “gradational worlds.” “In a big-class world, inequalities in life conditions are organized around such familiar sociological categories as professional, manager, service worker, craft worker, and laborer (Weeden and Grusky, 2012).” This categorization can be thought of as occupational macroclasses. In comparison, “In a microclass world, the same forces of selection, training, interactional closure, interest formation, and learning generalization are at work, but they operate principally at the level of institutionalized occupations (Weeden and Grusky, 2012).” Moreover, in a gradational world, “the life chances, politics, and lifestyles of classes are defined by their position in a hierarchy indexed by class income or prestige. The mechanisms underlying a gradational world are very similar to those in play for our two class worlds, but now they take on a simplified form in which income or prestige considerations become the principal levers through which these mechanisms operate (Weeden and Grusky, 2012).”

Weeden and Grusky (2012) also argue that big classes are simplifying into economic aggregates that can be better represented gradationally based on income. Indeed, other research has found that occupational macroclasses are declining in cohesion in other respects. Cheng and Park (2020) found that while the boundaries between sets of occupations are becoming increasingly rigid, these boundaries are increasingly decoupling from macroclasses and becoming a product of skill requirements. Other research argues that attributes separate from class—such as the degree of autonomy or specialized training a job requires—have profound, otherwise unexplained impacts on intergenerational mobility (Hout, 1984).

Separate from class analysis, everyday mobility patterns have rapidly expanded as an area of study within the broader field of social stratification. Some studies have found that the relations between neighborhoods are predictive of many adverse neighborhood outcomes, such as COVID-19 incidences, homicides, fatal police shootings, poor child health, medical emergencies, and adverse birth outcomes (Levy et al., 2020; Levy et al., 2022; Vachuska and Levy, 2022a; Vachuska and Levy, 2022b; Vachuska, 2023a, 2023b; Candipan et al., 2025). Indeed, theory strongly suggests that exposure to such incidents can greatly impact one’s life chances through many of the same mechanisms as traditional neighborhood effects.

In this paper, I analyze occupational inequality across a relatively-unexplored domain: everyday mobility patterns. Based on a review of the literature, I argue that occupation is an important predictor of mobility patterns. In an empirical analysis, I explore the predictive power that occupational class accords to everyday mobility patterns. I find that occupational class is generally one of the most meaningful demographic characteristics for predicting mobility patterns. I subsequently analyze the association between neighborhood occupational composition and exposure to poverty via everyday mobility patterns. With a battery of controls, I find that divides exist between occupational classes that are perceived as being of similar status or even grouped together entirely. For example, neighborhoods with more residents in professional occupations are associated with more visits to impoverished neighborhoods compared to neighborhoods with fewer residents in managerial occupations. Similarly, although individuals working in food preparation receive far less pay than individuals working in farming, fishing, and forestry, the latter are exposed to neighborhood poverty far more. I also discuss common threads in the patterns of exposure to poverty in the everyday mobility patterns that I observe.

The remainder of the paper is arranged as follows. First, I provide a brief overview of the literature regarding *occupational class analysis* and *neighborhood mobility patterns.* I subsequently describe the data I use to explore the relationship between the two areas and the methods I engage in. I then present the results and a discussion of the implications of this work.

### Occupational class

Sociologists have long been interested in studying social stratification through the lens of occupation, as it is strongly linked to life chances and is thus a straightforward variable by which to analyze both intra- and inter-generational mobility (Featherman and Hauser, 1976; Hauser and Warren, 1997).

Historically, many sociologists have recognized the importance of occupation in shaping social inequality. Marx divided society into two broad classes: the bourgeoisie and the proletariat, distinguishing these classes according to who controlled the means of production. Marx later described two additional classes: the petty bourgeoisie and the lumpenproletariat (Marx et al., 2019). Subsequent Marxist scholars have described other classes, such as the professional-managerial class (Horton, 1979). Occupation has also been central to other classic sociological theories. For example, Durkheim posited that occupation is central to social solidarity and conflict. More recently, Bourdieu (2018) concept of cultural capital highlights how an individual’s occupation shapes their social position and ability to procure resources.

Ultimately, what makes occupational class meaningful has varied substantially within sociology. During the growth of social stratification scholarship in the 20th century, scholars’ interest in occupational class was central to understanding stratification processes—both intra- and inter-generational—in terms of how immobile one’s life chances are with a given occupation.

Twentieth-century research on social stratification focused on the levels of education and income associated with specific occupations, assigning them socioeconomic scores based on these two factors. However, more recent research has argued that measures of class attainment must be more specific and context-dependent (Hauser and Warren, 1997). There has also been debate in the literature about how best to group occupations, with some scholars advocating for broad macro-classes based on an individual’s position in the capitalist arrangement of occupations. In contrast, others have suggested breaking occupations into smaller categories or using gradational measures such as income or prestige. Recent research has suggested that the significance of occupational macro-classes may be declining, with some scholars arguing that income and other job-specific attributes, such as autonomy and specialized training, are becoming more important in determining mobility (Hout, 1984; Weeden and Grusky, 2012; Cheng and Park, 2020).

Despite the decline in class analysis, occupation remains a critical lens through which sociologists understand social stratification. Occupation is strongly linked to an individual’s social position and the resources they can access. Those in high-status occupations tend to have disproportionately higher wages and better working conditions than those in low-status occupations. In an increasingly polarized labor market, this is true now more than ever (Hodson and Kaufman, 1982; Sakamoto and Chen, 1991; Kearney, 2014; Piore, 2018). Occupation is also tied to education and training, as certain occupations require more education, specialized training, or licensure than others. This adds additional barriers to entry and further perpetuates social inequality (Weeden, 2002). In addition to being tied to an individual’s social position and resources, occupation is also tied to cultural capital, among other non-economic resources that contribute to social mobility. Those in high-status occupations are disproportionately more likely to have access to networks and social connections that can help them advance in their careers (Oesch and Von ow, 2017; Cheng and Park, 2020).

Broadly, however, one central mechanism by which occupation stratifies American society intragenerationally is through the unequal distribution of income and wealth. Research has shown that individuals in higher-paying occupations, such as management and professional occupations, tend to have substantially higher levels of income and wealth than those in lower-paying occupations, such as service and manual labor (Korpi and Palme, 1998; Western et al., 2012). This unequal distribution of income and wealth can lead to differences in access to resources such as education, housing, and healthcare, which can further reinforce social stratification and further perpetuate intergenerational patterns. For example, research suggests that a higher income enables greater investment in children, which has substantial consequences for human capital outcomes (Kaushal et al., 2011; Mazumder and Davis, 2011; Dahl and Lochner, 2012; Corak, 2013; Kornrich and Furstenberg, 2013; Duncan et al., 2017).

Occupational attainment is also a strong signal of the unequal distribution of cognitive and non-cognitive skills. Research has shown that individuals in higher-paying occupations tend to have higher cognitive and non-cognitive skills than those in lower-paying occupations (Heckman et al., 2006; Coverley, 2006). Some research suggests that this causal relationship between personality and occupation goes both ways (Kohn and Schooler, 1982). This unequal distribution of cognitive and non-cognitive skill requirements can lead to differences in access to resources such as education, housing, and healthcare, further reinforcing social stratification. These inequalities in income and wealth also have consequences for intergenerational mobility. Research suggests that key cognitive and non-cognitive skills are strongly transmitted across generations (Duncan et al., 2017; Anger, 2012).

### Everyday mobility patterns

Everyday mobility patterns have garnered significant attention in sociology research in recent years. According to Wang et al. (2018), everyday mobility patterns are surprisingly stable, and people visit a wide variety of neighborhoods near or far from their own in everyday life. This work builds on earlier urban sociology research demonstrating that a neighborhood’s degree of isolation plays a key role in its vitality (Wilson, 2012). In other words, it is argued that visitors largely influence the everyday behaviors, interactions, and activities within a neighborhood. For example, Levy et al. (2020, 2022) found that the level of disadvantage within a neighborhood’s mobility network is a more accurate predictor of the neighborhood’s level of violence and adverse public health outcomes than the level of disadvantage among the neighborhood’s residents. Similarly, Graif et al. (2021) observed that neighborhoods with higher levels of mobility isolation in Chicago tended to have higher rates of violent crime.

Previous studies have shown that ties are stronger between demographically and socioeconomically similar neighborhoods. Several studies of individual urban areas have found that social similarity and spatial proximity are the primary drivers of neighborhood connections (Hipp and Perrin, 2009; Schaefer, 2012). At a national level, Wang et al. (2018) found that stronger ties tend to exist between racially similar neighborhoods. Other research has also identified that visit homophily may persist across other neighborhood characteristics, such as crime (Bastomski et al., 2017).

The composition of neighborhood mobility patterns is a critical factor in determining neighborhood outcomes, such as violence, homicide, fatal police shootings, infectious diseases, child health, medical emergenices, and birthweight (Graif et al., 2021; Levy et al., 2020; Vachuska and Levy, 2022a; Levy et al., 2022; Vachuska and Levy, 2022b; Vachuska, 2023a, 2023b; Candipan et al., 2025). The main drivers of these outcomes are the socioeconomic status of the neighborhoods that residents visit and the socioeconomic status of visitors to the neighborhood (Levy et al., 2020).

While everyday mobility patterns are a novel lens through which scholars may examine neighborhood effects, studying residential neighborhood characteristics is a much older field of study (Shaw and Mckay, 1942). Neighborhoods are generally understood as a key mediator through which disadvantage is transmitted intergenerationally (Sharkey and Faber, 2014). Scholars generally agree that everyday mobility patterns matter for the same reasons that residential neighborhoods matter (Levy et al., 2020). As such, it is logical to conclude that everyday mobility patterns may be consequential for education, cognitive and non-cognitive skills, occupational attainment, long-term health, and many other outcomes that are causally driven by neighborhoods (Mouw, 2000; Sharkey and Elwert, 2011; Wodtke et al., 2011; Carlson and Cowen, 2014; Jargowsky, 2014; Chetty et al., 2015). At the very least, empirical evidence highlights that the residents of mobility-disadvantaged neighborhoods are disproportionately exposed to violence, homicide, fatal police shootings, infectious diseases, medical emergenices, poor child health, and adverse birth outcomes (Graif et al., 2021; Levy et al., 2020; Vachuska and Levy, 2022a; Levy et al., 2022; Vachuska and Levy, 2022b; Vachuska, 2023a, 2023b; Candipan et al., 2025).

### Why do these bodies of literature fit together?

Occupation is a focal lens through which sociologists understand social stratification. Occupation is both a cause and consequence of life’s (dis)advantages. Those in the highest-status occupations enjoy better life outcomes and typically have children that grow up to live more successful lives (Featherman and Hauser, 1976; Hauser and Warren, 1997). Beyond causality, occupation also serves as a focal signal for other mechanisms of stratification, such as educational attainment and cognitive and non-cognitive skills (Kaushal et al., 2011; Mazumder and Davis, 2011; Dahl and Lochner, 2012; Corak, 2013; Kornrich and Furstenberg, 2013; Duncan et al., 2017).

Ultimately, occupation is a key driver of everyday mobility patterns. Trips to and from workplaces or work sites constitute a large share of activity patterns (Vagni and Cornwell, 2018). Some research has even utilized work-commuting patterns as a proxy for everyday mobility patterns in general (Graif et al., 2021). As such, it is reasonable that the locations and neighborhoods that residents of a particular neighborhood travel to for work constitute a major share of the neighborhood’s everyday mobility patterns. In addition, social ties generated from work may also reinforce these everyday mobility patterns (Feld, 1981).

Occupation is also a key lens through which to analyze intergenerational mobility. If occupation is associated with neighborhood mobility patterns, this may represent a new perspective through which occupation can stratify life outcomes, since strong evidence suggests that neighborhoods and everyday mobility patterns are important mechanisms by which disadvantage is transmitted intergenerationally. While demonstrating that mobility patterns mediate an important link between occupations and inter- or intra-generational outcomes is well beyond the scope of this paper, I do seek to demonstrate that occupations are a key predictor of everyday mobility patterns. Considering the robust and growing body of literature that indicates everyday mobility patterns are relevant for predicting a variety of neighborhood outcomes, this research highlight potentially stratifying neighborhood-occupation relationships. While research has highlighted that occupational classes are associated with various attributes that may reinforce intergenerational immobility, I seek to highlight everyday mobility patterns as an additional mechanism. To ensure clarity, I focus exclusively on the neighborhood-level associations between occupational composition and mobility patterns, without making claims about individual-level or intergenerational outcomes. This paper does not attempt to demonstrate causal relationships or to measure the specific impacts of mobility patterns on life trajectories. Instead, it provides a descriptive analysis that highlights how occupations may stratify access to neighborhood contexts through everyday mobility patterns, offering a foundation for future research to explore the implications of these associations.

## Data

Data for this project comes from three sources. First, data on mobility patterns was obtained from Safegraph’s “Weekly Patterns” dataset. Safegraph is a foot traffic data company that constructs a detailed mobility patterns database based on a panel of 45 million nationally representative mobile devices. The “Weekly Patterns” dataset contains data on the number of visits received by points of interest and the census block groups in which visitors reside. Residence is estimated based on the common nighttime location of the mobile device. Data for the 51^1^ weeks of 2019 are aggregated such that the number of visitors from census block group *i* to point of interest *j* are summed across all weeks. Metadata on points of interest was obtained from the second source, Safegraph’s “Point of Interest” dataset. Finally, I obtained demographic and socioeconomic data on census block groups from the American Community Survey’s 2015–2019 5-year estimates. Specifically, I obtained data on poverty rates, educational attainment, income distribution, age distribution, racial and ethnic composition, and occupational composition for all census block groups in the United States.

## Methods

### Categorical analysis

In the first analysis, I examine what variables are the best predictors of visits to specific points of interest. I test five models based on five sets of demographic and socioeconomic variables: occupational composition, age composition, income distribution, racial and ethnic composition, and educational attainment composition. For each approach, I estimate a Poisson model of the number of visits to a specific category of points of interest. The coefficients in each model include the number of individuals or households in each distribution category. The form of each model can be written as follows:


$$\ln\left(v_{ip}\right)=\beta_{c}*c_{i}+\nabla_{k}+\varepsilon_{i}$$

Where $v_{ip}$ represents the number of visitors from neighborhood *i* to any specific points of interest in category p, $c_{i}$ represents a vector of values depicting the number of individuals or households in each category in the distribution in neighborhood *i*, and $\nabla_{k}$ depicts a fixed effects term wherein *k* is the county in which neighborhood *i* is located. Model fit is evaluated using BIC and log-likelihood.

As a secondary exploration of whether occupation significantly contributes to the predication of mobility patterns, I estimate two additional sets of models. In the first set, I include age composition, income distribution, racial and ethnic composition, and educational attainment composition. In the second set, I add occupational composition. Based on changes in BIC and log-likelihood, I evaluate if the occupational composition improves the model’s fit.

### Poverty analysis

In the second analysis, I examine what variables predict visits to high-poverty neighborhoods. A recent body of research emphasizes the importance of mobility patterns to and from socioeconomically-disadvantaged neighborhoods as a leading cause of adverse neighborhood outcomes (Levy et al., 2022). In this analysis, I estimate the average out-degree of poverty, in line with recent methodology, using the following formula:


$$OPOV_{i}=\frac{\sum v_{ij}*POV_{j}}{\sum v_{ij}}$$

Where $v_{ij}$ represents the number of visits from census block group *i* to point of interest *j* during the year 2019. $POV_{j}$ represents the poverty rate of the census block group in which point of interest j is located. Subsequently, I estimate the following model:


$$OPOV_{i}=\beta_{c}*c_{i}+\nabla_{k}+\varepsilon_{i}$$

Where $c_{i}$ represents a vector of values depicting the number of individuals or households in each category in the distribution in neighborhood *i,* and $\nabla_{k}$ depicts a fixed effects term wherein *k* is the county in which neighborhood *i* is located.

It is important to acknowledge that the analysis conducted at the neighborhood level may suffer from the ecological fallacy. This limitation arises from the assumption that individual experiences align perfectly with the characteristics of the overall neighborhood. Although the models include neighborhood-level variables, such as occupational composition and poverty rates, they do not directly account for individual-level characteristics or experiences. Therefore, the findings should be interpreted as associations between neighborhood-level variables and outcomes rather than direct evidence of individual-level relationships. Despite this limitation, analyzing neighborhood-level data provides valuable insights into the predictors of mobility patterns and visits to specific types of points of interest. Future research that incorporates individual-level data would contribute to a more comprehensive understanding of the complex dynamics between individual characteristics, neighborhood factors, and mobility patterns.

## Results

### Overall category frequencies

Table 1 presents the results of the model tests. Rows one and two explore the model fit for all categories of points of interest and the most common (most visited quantile) without county-fixed effects. The results indicate that occupational composition fits best based on BIC and log-likelihood about 40% of the time for all categories and around 16% for the most common types of places. When considering the second-best fit, occupational composition fits best or second-best about 84% of the time for all places and 96% for the most common places. In terms of additive power, occupational composition improves full model BIC and log-likelihood more than 90% of the time.

**Table 1:** Predictive performance of occupational composition on visits to specific place categories.

	POIs	County fixed effects?	Best BIC?	Best Loglik?	Best or second best BIC?	Best or second best Loglik?	Improved BIC in full model	Improved Loglik in full model?	*N*
1	All	No	40.60%	41.40%	84.90%	84.90%	94.50%	94.80%	372
2	Most common	No	16%	16%	96%	96%	90.40%	90.40%	75
3	All	Yes	25.50%	23.90%	51.30%	51.60%	93.50%	99.70%	372
4	Most common	Yes	10.70%	10.70%	25.30%	25.30%	100%	100%	75

The county-fixed effects substantially weaken how often occupational composition produces the best or second-best model. However, it should be noted that occupational composition has much higher intercounty variation than other variables, so this is not surprising. Despite not always being the best-fitting model, occupational composition improves overall model fit more than 93% of the time for BIC and log-likelihood with county-fixed effects.

### Poverty

Table 2 reveals the results of the models of Out-degree of poverty (OPOV). Model one includes the percentage of employed individuals in each occupational class, with management, business, and financial operations occupations as the reference group. These results suggest that other occupational classes have a far higher OPOV than the managerial class. Even occupations with similar levels of status, such as professional occupations, have substantially higher rates of poverty. Model two adds a control for spatial lag, that is, the average poverty rate in the 10 nearest census block groups. Adding this substantially attenuates all of the occupational effects, suggesting part of the reason why neighborhoods with high proportions of individuals working in management, business, and financial operations occupations tend to not be located near neighborhoods with high poverty rates.

**Table 2:** OLS models predicting OPOV by neighborhood.

	Model 1	Model 2	Model 3
Spatial lag		0.503***	0.422***
		(0.001)	(0.001)
Professional	0.131***	0.031***	0.020***
	(0.002)	(0.001)	(0.001)
Healthcare support	0.281***	0.025***	−0.011***
	(0.003)	(0.002)	(0.002)
Protective service	0.288***	0.075***	0.044***
	(0.004)	(0.002)	(0.002)
Food prep. and serving	0.256***	0.014***	−0.022***
	(0.002)	(0.001)	(0.001)
Building and grounds	0.270***	0.018***	−0.026***
	(0.003)	(0.002)	(0.002)
Personal care	0.157***	0.005*	−0.023***
	(0.003)	(0.002)	(0.002)
Sales	0.161***	0.029***	−0.002
	(0.002)	(0.001)	(0.001)
Office and admin.	0.147 ***	0.025***	−0.007***
	(0.002)	(0.001)	(0.001)
Farming, fishery, forestry	0.295***	0.083***	0.043***
	(0.004)	(0.002)	(0.002)
Construction	0.136***	0.028***	−0.003**
	(0.002)	(0.001)	(0.001)
Production	0.206***	0.035***	0.008***
	(0.002)	(0.001)	(0.001)
Transportation	0.194***	0.013***	−0.015***
	(0.002)	(0.001)	(0.001)
Controls			X
*N*	218,588	218,588	218,588
AIC	1361638.677	1127117.381	1103684.431
BIC	1361772.511	1127261.510	1104353.602
Adj. R^2^	0.197	0.725	0.753

***p < 0.001; **p < 0.01; *p < 0.05.

**Figure 1:**
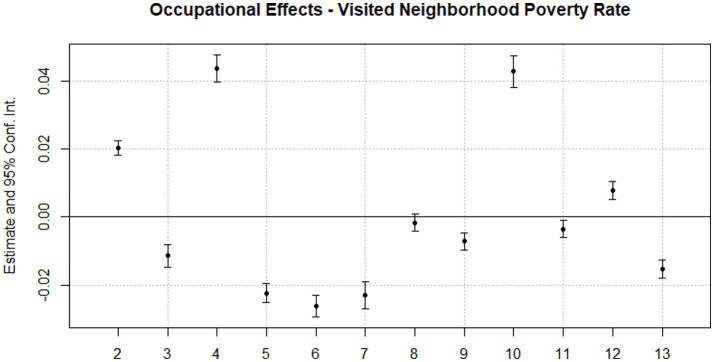
Occupational effects-visited neighborhood poverty rate. (1) Management, business, and financial operations (reference), (2) professional, (3) healthcare support, (4) protective service, (5) food prep. and serving, (6) building and grounds, (7) personal care, (8) Sales, (9) office and admin., (10) farming, fishery, forestry, (11) construction, (12) production, (13) transportation.

Finally, model three adds controls for age composition, educational attainment composition, income composition, and racial and ethnic composition. These controls further attenuate occupational effects, even making the coefficient negative in a few cases. Ultimately, four occupational categories retain significant positive coefficients relative to the management, business, and financial operations reference group: professional occupations; protective service occupations; farming, fishing, and forestry occupations; and production occupations. Conversely, seven occupational categories have significant negative coefficients: healthcare support occupations, food preparation and serving occupations, building and grounds maintenance occupations, personal care occupations, office and administrative occupations, construction occupations, and transportation occupations. Ultimately, these results suggest that, net of a battery of controls, substantial distinctions exist between the neighborhoods that workers of different occupational classes visit. For example, a neighborhood that is 100% professional occupations would be expected to visit neighborhoods with an additional 2% of residents in poverty, net of the poverty rate in nearby neighborhoods and residents’ ages, educational attainment, income levels, and racial and ethnic compositions, relative to a 100% managerial occupations neighborhood. Figure 1 presents a visualization of the results of model three.

Ultimately, these models reinforce one historical thread related to occupational inequality and three novel threads. First, model one supports occupational socioeconomic status being strongly associated with advantage. The reference category, management, business, and financial operations occupations, experiences the lowest OPOV. Professional occupations experience the second-lowest OPOV. Both of these groups constitute occupations typically associated with the highest socioeconomic standing (Duncan, 1961). Individuals in these occupations are well-educated and have high incomes. Therefore, their exposure to low OPOV is at least partially a consequence of residing in more affluent neighborhoods.

Models two and three depend on the poverty rate of nearby neighborhoods, thus revealing the novel threads. First, occupational classes that predominately serve the middle or upper class are exposed to less OPOV than one might expect based purely on the socioeconomic status of the occupation. Health care, food preparation and serving, building and grounds maintenance, and personal care occupations all have relatively low exposure to poverty and are all closely tied to the care and service economies. Construction occupations, which are overwhelmingly concentrated in affluent areas, are also associated with relatively low exposure to poverty.

A second novel thread suggests the opposite tendency: occupations targeted at the disadvantaged tend to have greater than expected exposure to poverty. Protective service occupations are the central example of this. The greatest number of policing and security guard jobs are in disadvantaged neighborhoods. This trend is made clear by the strong association between protective service occupations and exposure to poverty in models two and three.

Finally, a third novel thread suggests industries that tend to be geographically marginalized are closely tied to greater-than-expected exposure to poverty. Models two and three indicate that farming, fishing, and forestry occupations and production occupations are exposed to higher OPOV. Both of these industries tend to be relegated to more disadvantaged areas due to “not in my backyard” preferences. These “not in my backyard” preferences reflect a common resistance by individuals or communities to have industries with potential negative impacts located near their homes, resulting in the concentration of these industries in economically marginalized areas. Additionally, both sets of occupations correlate with industries that may involve greater pollution and visual blight (Pol et al., 2006). As such, more industrial infrastructure tends to be present in rural or disadvantaged areas (Pastor et al., 2001).

It is important to acknowledge the limitations of interpreting the results presented in this analysis, particularly with respect to the ecological fallacy. The analysis conducted at the neighborhood level, while providing valuable insights into the associations between occupational composition and exposure to poverty in everyday mobility patterns, does not capture individual-level variation that may exist within neighborhoods. Thus, caution should be exercised in making direct inferences about individual experiences based solely on neighborhood-level associations. Future research should aim to examine these relationships at the individual level to obtain a more nuanced understanding of the complex dynamics between occupation, mobility patterns, and poverty exposure. By considering individual-level variation, I can better account for the diverse experiences within neighborhoods and gain a deeper understanding of how occupational class shapes individuals’ lived experiences of neighborhood poverty.

## Discussion

In this study, I have examined the relationship between occupational composition and everyday mobility patterns. The predictive results demonstrate that occupational composition fits best based on BIC and log-likelihood for about 40% of all categories and 16% of the most common types of places without county-fixed effects across a host of five categorical demographic predictors. Generally, neighborhood age composition is a better predictor of specific everyday mobility patterns than occupation. As a result, occupational composition was found to be the best or second-best for 84% of all point of interest types and 96% of the most common types. Moreover, in terms of additive power, occupational composition was found to improve BIC and log-likelihood more than 90% of the time.

The results of the OPOV models demonstrate that, net of neighborhood placement, exposure to poverty in everyday mobility patterns varies considerably with occupation and that the addition of controls for age composition, educational attainment composition, income composition, and racial and ethnic composition only slightly attenuates these effects. These results suggest that there are substantial distinctions between the types of neighborhoods visited by workers of different occupational classes.

I broadly group the implied results of the OPOV models into four threads. First, models that do not control for the context proximal to neighborhoods suggest that classic measures of socioeconomic status (such as education and income) predict exposure to poverty in everyday mobility patterns. This is because individuals working in higher socioeconomic positions have greater neighborhood attainment and tend to live in areas with lower poverty rates. Classical theory on social stratification supports this finding (Wilson, 2012).

The subsequent threads are more novel and pertain to the association between occupation and everyday exposure to poverty, net of the surrounding context. The second thread I identify is that occupations related to the service and care economies are better insulated from poverty. This can be understood as a consequence of workers in these occupations primarily serving the middle and upper classes and subsequently having disproportionate contact with both affluent individuals and affluent neighborhoods. The third thread indicates the opposite: occupations directed at more disadvantaged populations experience greater exposure to poverty in everyday mobility patterns. A central example of this is protective service occupations, such as policing, which is disproportionally concentrated in impoverished and non-white neighborhoods (Makowsky et al., 2019; Gordon, 2020). The fourth thread pertains to the spatial marginalization of certain occupations. Research on “not in my backyard” preferences suggests that industries producing disproportionate amounts of air and noise pollution and visual blight are likely to be excluded from more affluent neighborhoods (Pol et al., 2006). I find support for this idea due to farming, fishing, forestry, and production occupations experiencing some of the greatest associations with everyday exposure to poverty.

Ultimately, the results of these models suggest that there are significant distinctions between the types of neighborhoods that workers of different occupational classes visit, even when controlling for a range of other factors, including age composition, educational attainment, income, and race and ethnicity. These findings contribute to a broader understanding of social stratification by showing that occupations are associated with different types of neighborhoods and, by extension, different types of social environments. A growing body of research on neighborhood effects suggests that this everyday exposure to poverty is strongly correlated with various adverse outcomes. Overall, these results provide suggestive evidence of further mechanisms through which social stratification may be reproduced and perpetuated through the occupational structure. However, it is important to emphasize that these findings are descriptive in nature and focus solely on neighborhood-level patterns. This study does not establish causal relationships between occupational mobility patterns and individual or intergenerational outcomes. Rather, it highlights the potential role of everyday mobility patterns as a stratifying mechanism at the neighborhood scale. By demonstrating these associations, this work lays the groundwork for future studies to investigate how occupationally linked mobility patterns might interact with other forms of neighborhood disadvantage and contribute to broader processes of social stratification.

There are several limitations to interpreting the results presented in this paper. First, the models only explore the relationship between occupational composition and visit patterns and do not necessarily reflect a causal relationship or have a clear mechanism. While there is no clear omitted variable, it is possible that other unmeasured factors may drive both occupational composition and the frequency of visits to points of interest. An additional limitation is that while disadvantage in mobility patterns has been demonstrated to be important for understanding adverse neighborhood outcomes, the finding that occupational composition is associated with neighborhood mobility patterns does not necessarily imply that occupational composition is associated with the adverse outcomes that tend to be associated with neighborhood mobility patterns. Future research is needed to further test such an assumption.

Furthermore, the interpretation of the results is limited by the ecological fallacy. By examining relationships at the neighborhood level, one may be inclined to assume that individual experiences align with the characteristics of the neighborhood. However, individual-level differences and experiences may exist, potentially leading to discrepancies between the neighborhood-level analysis and individual experiences. Therefore, it is crucial to interpret the findings as associations between neighborhood-level variables and outcomes rather than direct evidence of individual-level relationships.

Future research could build upon the findings of this study in several areas. It would be useful to further explore the mechanisms through which occupation influences everyday mobility patterns. Future research could investigate whether certain occupations have different levels of access to transportation or leisure time, which may influence the types of places they can visit. Similarly, examining whether certain occupations are more likely to generate social connections to certain neighborhoods would provide insight into their visitation patterns. Descriptive analyses that measure travel-time adjusted visit ratios or estimate occupational composition in daily environments, may also shed light on the nature of occupational correlates with mobility patterns (Vachuska, 2023a, 2023b; Vachuska, 2024). Moreover, future research could evaluate the potential for interventions to mitigate the experienced poverty in neighborhoods visited by workers of different occupational classes. Policies that promote affordable housing or increase access to education may help to reduce everyday poverty and improve neighborhood quality for workers in various occupations. Further investigation into the threshold effects of neighborhood composition and policies emphasizing the integration of different socioeconomic groups would also be valuable.

In summary, while this study contributes to an understanding of the association between occupational composition and mobility patterns, it is important to consider the aforementioned limitations and the need for further research to provide a comprehensive understanding of the complex dynamics between occupation, mobility, and neighborhood experiences.

## Data Availability

The data analyzed in this study is subject to the following licenses/restrictions: data for this project was obtained from SafeGraph and cannot be reshared per the terms of service of the original data license. Requests to access these datasets should be directed to https://www.safegraph.com/.

## Footnotes

^1^I include 51 rather than 52 weeks because the 52nd week extends into 2020.
